# Fetal bladder rupture in posterior urethral valves: a clinically relevant complication or a protective pop-off mechanism

**DOI:** 10.3389/fped.2026.1880245

**Published:** 2026-07-10

**Authors:** Karolina Krzywiecka, Natalia Lekston, Zofia Sieroń, Hanna Kubik, Agnieszka Wiernik, Grzegorz Kudela

**Affiliations:** 1 Students’ Scientific Club at the Department of Pediatric Surgery and Urology, Faculty of Medical Sciences in Katowice, Medical University of Silesia, Katowice, Poland; 2 Department of Pediatric Surgery and Urology, Medical University of Silesia, Katowice, Poland

**Keywords:** bladder augmentation, pop-off mechanisms, posterior urethral valves, urinary ascites, urinary bladder rupture

## Abstract

**Introduction:**

Posterior urethral valves (PUV) are the most common cause of congenital bladder outlet obstruction in male infants and may result in kidney damage and chronic kidney disease. In severe cases, spontaneous fetal bladder rupture with urinary ascites may occur. This study presents a rare case of fetal bladder rupture secondary to PUV requiring complex bladder reconstruction and systematically reviews similar cases.

**Methods:**

We report a male patient with prenatal bladder rupture due to PUV. A systematic review was conducted according to PRISMA guidelines using PubMed, Embase, Scopus, Web of Science, and the Cochrane Library up to March 2026. Pediatric cases of bladder rupture associated with PUV were included.

**Results:**

Prenatal imaging showed bladder rupture with urinary ascites at 23 weeks’ gestation. Postnatal treatment included endoscopic valve ablation, vesicostomy, and subsequent ileocystoplasty with a continent catheterizable channel using the Macedo technique. Kidney function remained stable, but severe bladder dysfunction required reconstruction. The review identified 15 patients with PUV-associated bladder rupture. Prenatal diagnosis was reported in 33% of cases, most often in the third trimester. Fetal ascites, hydronephrosis, and oligohydramnios were the main prenatal findings. Management usually involved urgent postnatal urinary decompression followed by valve ablation. Follow-up was limited, but available reports suggested relatively preserved kidney function and frequent bladder dysfunction.

**Discussion:**

Fetal bladder rupture in PUV is a rare and severe manifestation of lower urinary tract obstruction. Although proposed as a potential pressure-relieving “pop-off” mechanism, this hypothesis remains speculative and unsupported by robust evidence. Available data are limited to heterogeneous case reports. Multidisciplinary follow-up is essential to assess kidney and bladder outcomes in this group of patients.

**Systematic Review Registration:**

PROSPERO registration: CRD420261367565.

## 1 Introduction

Congenital anomalies of the lower urinary tract represent an important group of urological conditions in the pediatric population and may lead to significant impairment of kidney and bladder function. One of the most common causes of congenital bladder outlet obstruction are posterior urethral valves (PUV), occurring with a frequency of approximately 1 in 7,000–8,000 live births. PUV causes obstruction of urinary outflow and increased intravesical pressure beginning in fetal life, which may result in progressive kidney damage and, in some patients, chronic kidney disease (CKD) (1, 2).

Spontaneous fetal bladder rupture is a rare and severe manifestation of lower urinary tract obstruction in PUV. It may present with urinary ascites and reflects markedly increased intravesical pressure during fetal development. Bladder rupture should not be regarded as a benign event. It may be associated with abnormal bladder development and subsequent bladder dysfunction, including reduced capacity, poor compliance, overactivity and urinary incontinence (1, 3). Some patients with severe persistent dysfunction may require reconstructive surgery. The aim of reconstruction is to create a larger, low-pressure reservoir to protect the upper urinary tract.

In response to elevated pressure, so-called “pop-off” mechanisms have been described as potential pathways for decompression of the urinary tract. These include rupture of the calyceal fornices, bladder diverticula, unilateral high-grade vesicoureteral reflux (VUR) associated with a hypoplastic or dysplastic kidney (VURD syndrome) and persistent urachal patency (4). Although spontaneous bladder rupture has also been hypothesized to represent an extreme pressure-relieving “pop-off” mechanism, any potential renoprotective effect remains speculative and is not supported by robust clinical evidence (1).

The aim of this study is to present a rare case of prenatal bladder rupture secondary to PUV and to provide a systematic review of the literature on this rare and severe complication, with attention to clinical presentation, management, and renal and bladder outcomes.

## 2 Materials and methods

### 2.1 Case report

Medical records of a patient with prenatal bladder rupture and urinary ascites secondary to PUV, diagnosed and treated at our medical center, were retrospectively reviewed. The study was conducted in accordance with the Declaration of Helsinki and approved by the Bioethics Committee of our University. Written informed consent for publication was obtained from the patient and his parents.

Clinical data, including prenatal findings, postnatal course, surgical management, and follow-up outcomes, were collected and analyzed to provide a comprehensive description of the case.

### 2.2 Systematic review

This systematic review was conducted in accordance with the Preferred Reporting Items for Systematic Reviews and Meta-Analyses (PRISMA) guidelines (5). The review question was structured using the PICO framework:

Population: Pediatric patients, including fetal, neonatal, infant, and childhood cases, diagnosed with PUV complicated by bladder rupture, with or without urinary ascites.

Intervention/Exposure: Prenatal or postnatal management of PUV-associated bladder rupture, including fetal interventions such as fetocentesis or shunt placement, and postnatal interventions such as bladder catheterization, peritoneal drainage, valve ablation or incision, vesicostomy, surgical repair of bladder rupture, urinary diversion, and reconstructive bladder surgery.

Comparator: Not applicable, as the available evidence consists predominantly of descriptive case reports and no control group was expected.

Outcomes: Timing and method of diagnosis, prenatal and postnatal imaging findings, type of prenatal and postnatal intervention, complications, survival, renal outcomes, bladder function, continence status, and need for reconstructive surgery.

Study design: Case reports, case series, and observational studies reporting pediatric cases of bladder rupture in the context of PUV were considered eligible for inclusion.

The review was registered in the International Prospective Register of Systematic Reviews (PROSPERO; registration number: CRD420261367565), and the full protocol is provided as Supplementary Material.

### 2.3 Search strategy

A comprehensive search of PubMed, Embase, Scopus, Web of Science, and the Cochrane Library was performed using the following terms: (“posterior urethral valves” OR “PUV” OR “LUTO”) AND (“bladder rupture” OR “urinary ascites” OR “urinoma” OR “pop-off”). The search strategy was adapted for each database as appropriate. No date restrictions were applied, and all studies published up to March 23, 2026 were included.

### 2.4 Eligibility criteria

Studies reporting pediatric cases of bladder rupture with/without urinary ascites in the context of PUV were included. Exclusion criteria comprised non-English publications, conference abstracts, video materials, letters to the editor, reviews, and studies with incomplete or duplicate data.

### 2.5 Study selection

**Figure 1:**
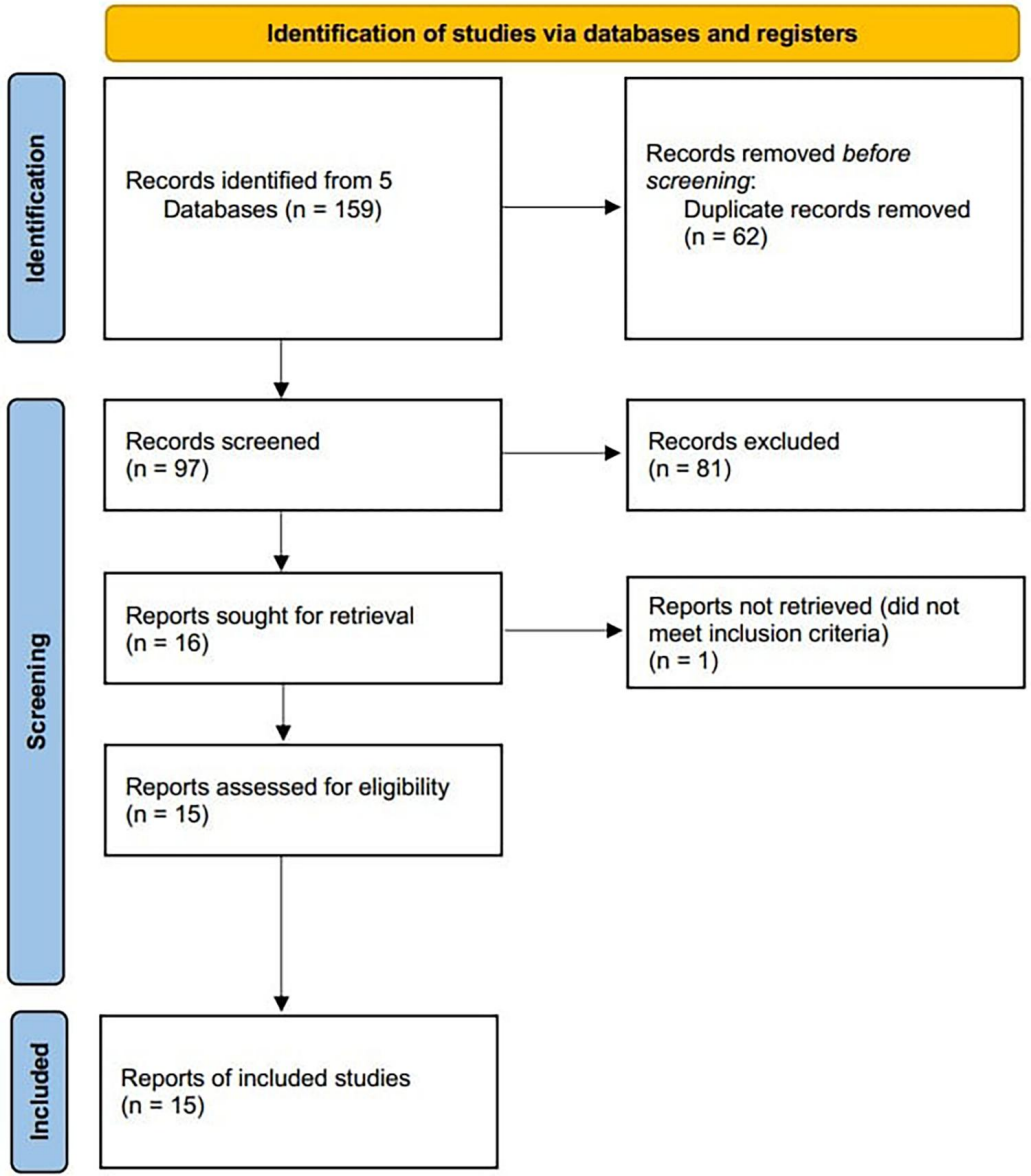
PRISMA flow diagram for literature review.

After removal of duplicates, three reviewers (KK, NL, ZS) independently screened titles and abstracts. Full-text articles were subsequently assessed for eligibility. Any disagreements were referred to a fourth reviewer (AW) and, when needed, resolved through discussion and consensus within the research team. The study selection process is shown in the PRISMA flow diagram (Figure 1).

### 2.6 Risk of bias assessment

The risk of bias of included studies was assessed independently by two reviewers (KK, NL) according to study design. As all included studies were single case reports, they were evaluated using the Joanna Briggs Institute (JBI) critical appraisal checklist (6) for case reports. Each item was rated as “yes,” “no,” or “unclear,” and an overall quality rating (low, moderate, or good) was assigned based on the proportion of criteria met.

Any disagreements were resolved through consultation with a third reviewer (ZS) or, when necessary, by discussion and consensus within the research team. The results of the risk-of-bias assessment were considered in the interpretation of the findings. Given the predominance of descriptive study designs, the overall level of evidence is inherently limited and should be interpreted with caution.

### 2.7 Data extraction and analysis

Data were extracted into a predefined database (Microsoft Excel 2007, Redmond, WA, USA), including study characteristics (author, year, and setting) and patient-level variables: age at diagnosis, gestational age, timing of diagnosis, prenatal imaging findings, clinical presentation, diagnostic methods, interventions, complications, and outcomes, including survival and kidney function. Owing to substantial heterogeneity in study design, outcome definitions, and reporting, data were synthesized descriptively and narratively according to study design, population characteristics, diagnostic approaches, interventions, and outcomes.

## 3 Results

### 3.1 Case report

During a prenatal ultrasound (US) examination at 22 weeks of gestation, a male fetus was found to have bilateral dilatation of the upper urinary tracts and an enlarged bladder. On follow-up imaging at 23 weeks of gestation, a large amount of fluid was visualized within the fetal peritoneal cavity, accompanied by an empty bladder and non-dilated upper urinary tracts, suggesting bladder perforation with urine extravasation into the peritoneum.

Because of severe oligohydramnios, amnioinfusion was initially performed to restore amniotic fluid volume. The exact volume of fluid infused and the detailed criteria used to assess the adequacy of amnioinfusion were not available in the retrospective documentation. During fetal intervention, the bladder was collapsed and could not be reliably visualized, whereas a large amount of intraperitoneal fluid consistent with urinary ascites was present. Therefore, vesicoamniotic shunting was not feasible, and a peritoneoamniotic shunt was placed to drain the urinary ascites. Additionally, antenatal steroid therapy was administered to promote fetal lung maturation. Subsequent prenatal US demonstrated recurrent dilatation of the upper urinary tracts and a stiff, thick-walled, collapsed bladder.

**Figure 2:**
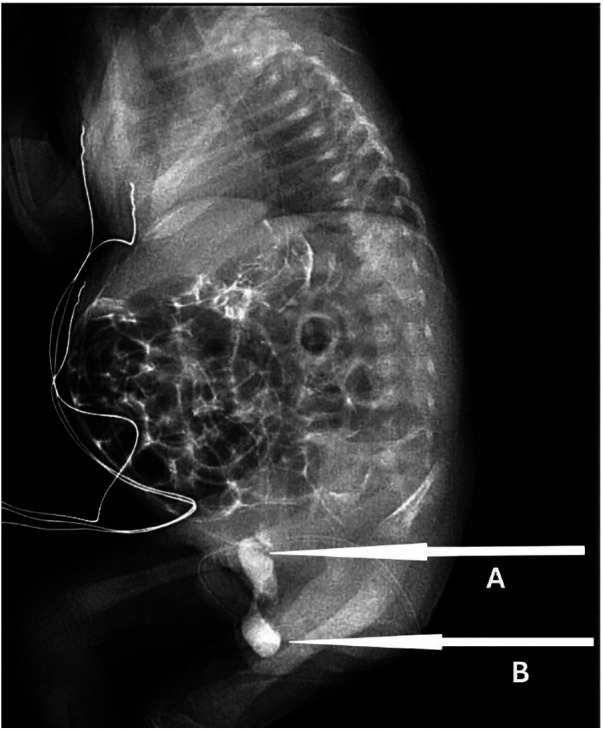
Voiding cystourethrogram (VCUG) demonstrating intravesical contrast administered via catheter, visualized between bowel loops on lateral projection. **A**) bladder rupture, **B**) posterior urethra.

The child was delivered at 32 weeks of gestation via cesarean section, with a birth weight of 1820g and Apgar scores of 5 and 8. After birth, a bladder catheter was inserted, achieving effective urinary drainage. Voiding cystourethrogram (VCUG) demonstrated leakage of contrast from the bladder into the peritoneal cavity and showed a dilated posterior urethra, consistent with PUV (Figure 2). On the 5th day of life, renal US revealed significant urinary stasis and a thickened bladder wall.

In the eighth week of life, cystoscopy was performed and the PUV were incised using a cold knife. A vesicostomy was then created at the site of bladder perforation, providing effective urinary drainage.

During nephrology follow-up, normal kidney function and normal upper urinary tract anatomy were observed. Urodynamic evaluation at the age of five years revealed reduced bladder capacity and poor compliance. Six months of treatment with solifenacin did not result in significant improvement. Therefore, the patient underwent ileocystoplasty for bladder augmentation with simultaneous creation of a continent catheterizable channel using the Macedo technique.

On the seventh postoperative day, the patient required laparoscopic adhesiolysis because of adhesive small-bowel obstruction near the anastomosis with the augmented bladder. These adhesions were successfully released during a laparoscopic procedure performed on the seventh postoperative day. The subsequent postoperative course was uneventful, and the patient was discharged home in good condition on the tenth postoperative day.

The child continues to be regularly monitored in both urology and nephrology clinics. Kidney function was monitored using serial serum creatinine measurements and estimated glomerular filtration rate (GFR). The latest serum creatinine level was 0.55 mg/dL, corresponding to an estimated GFR of approximately 96 mL/min/1.73 m^2^ according to the Schwartz formula. During two years of follow-up after bladder augmentation, kidney function remained stable, upper urinary tract US findings were normal, and no symptomatic urinary tract infections were reported. Parents performed clear intermittent catheterization every three hours via the Macedo channel, with a nighttime break. The patient subsequently learned self-catheterization. Currently, at 8 years of age, the boy attends school and independently empties his bladder via catheterization through the channel, maintaining continence between catheterizations.

### 3.2 Systematic review

Of the 159 records identified (Cochrane: 2, PubMed: 23, Scopus: 43, Embase: 52, Web of Science: 39), 62 duplicates were removed, 97 articles were screened and 81 studies were excluded. Sixteen articles underwent full-text review, with one excluded after detailed assessment. Reference lists of eligible studies were also manually screened to identify additional relevant publications. Ultimately, 15 studies describing 15 patients with PUV complicated by bladder rupture were included (1, 3, 7–19).

The characteristics of the included studies the key clinical characteristics of the analyzed patients are summarized in Table 1.

**Table 1:** Key characteristics of the included single case reports; GA, gestational age; PUV, posterior urethral valve; VCUG, voiding cystourethrogram; IPV, intravesical pressure; US, ultrasound; MRI, magnetic resonance imaging; DTPA, diethylenetriamine pentaacetic acid; DMSA, dimercaptosuccinic acid; EDTA, ethylenediaminetetraacetic acid; MAG3 scan, mercaptoacetyltriglycine renal scintigraphy; x-ray, X-radiation.

Patient	Authors, year and country	Age at diagnosis	Age at PUV ablation	Prenatal interventions	Postnatal interventions	Postnatal examinations	Follow-up data	Quality Assessment (JBI)
1	Johanson et al., 1982, Sweden (7)	21 days of life	at 2 weeks of life	none	1. Surgical repair of bladder rupture 2. Resection of necrotic bladder dome 3. Suprapubic catheter 4. Temporary bladder fistula (cystostomy) 5. PUV ablation 6. Closure of cystostomy	1. VCUG 2. IPV	no data	Moderate
2	Chen et al., 1997, Taiwan (8)	2 days of life	at 2 weeks of life	1.Abdominal fetocentesis 2.Aspiration of urinomas	1. Urethral catheterization 2.Endotracheal intubation 3. Peritoneal drainage 4. PUV ablation	1. VCUG 2. US 3. MRI	no data	Moderate
3	Arora et al., 2001, India (9)	2–4 weeks of life	scheduled for a later time	none	1. Urethral catheterization 2. Antibiotics 3. Peritoneal drainage 4.Vesicostomy	1. US 2. VCUG	Regular monthly follow-up with normal DTPA renal scan; the infant is thriving with normal clinical and laboratory findings, although the exact duration of follow-up is not specified.	High
4	Lacher et al., 2006, Germany (10)	2 months	at 4 months of life	1. Abdominal fetocentesis 2.Peritoneoamniotic shunt	1. Peritoneal drainage 2. Suprapubic catheter 3. PUV ablation	1. US 2. VCUG	no data	High
5	Bataille et al., 2007, Belgium (11)	II trimester	at 9 days of life	none	1. Urethral catheterization 2. PUV ablation	1. VCUG 2. US 3. Isotopic nephrogram	1 year follow-up: normal micturitions, normal nephrogram	High
6	Aslam et al., 2007, USA (12)	13 days of life	scheduled for a later time	none	1. Urethral catheterization 2. PUV ablation 3. Vesicostomy	1. US 2. VCUG	no data	Moderate
7	Binkhorst et al., 2010, Netherlands (13)	III trimester	at approximately a few days after birth	none	1. Urethral catheterization 2. Antibiotics 3. PUV ablation 4. Surgical resection of prevesical cyst 5. Surgical repair of bladder	1. VCUG 2. MAG3 scan	2 months follow-up: creatinine was normal, urethrocystoscopy showed no residual valves, and MAG3 scan showed preserved renal function with delayed drainage due to hydronephrosis.	High
8	Gupta et al., 2013, India (14)	5 days of life	no data	none	1. Vesicostomy 2. Percutaneous nephrostomy 3. Bilateral loop ureterostomies	1. VCUG 2. US	no data	Moderate
9	Singh et al., 2013, India (15)	III trimester	no data - child died	1. Peritoneoamniotic shunt	Neonate died within hours of birth.	Neonate died within hours of birth.	Neonate died within hours of birth.	High
10	Oki et al., 2016, Japan (16)	21 days of life	at 21 days of life	none	1. Urethral dilation with progressively larger catheters 2. Urethral catheterization 3. Laparoscopic repair of the bladder 4. PUV ablation	1. VCUG	no data	Moderate
11	Magawa et al., 2018, Japan (17)	postnatally (unspecified)	at 7 months of life	1. Abdominal fetocentesis	1. PUV ablation	1. VCUG 2. US	At 7 months of follow-up, the infant showed adequate weight gain and underwent cystoscopic incision of the urethral valves.	High
12	Garriboli et al., 2021, UK (18)	3 years 3 month	3 years, 3 months	none	1. Laparotomy 2. Suprapubic catheter 3. Vesicostomy, 4. Peritoneal lavage 5. Circumcision 6. PUV resection 7. Bladder diversion	1. Abdominal x-ray 2. US 3. VCUG 4.Video- urodynamics 5. DMSA	3 years of follow-up, stable renal function, no urinary tract infections reported.	High
13	Adriaenssens et al., 2023, Belgium (1)	III trimester	at 1 month of life	1. Abdominal fetocentesis 2. Peritoneoamniotic shunt	1. Resection of the omentum with subsequent closure of the shunt insertion 2. PUV ablation	1. VCUG 2. DMSA 3. EDTA	8-year follow-up: normal renal function and ultrasonographic findings; however, bladder dysfunction persisted, manifested as overactive bladder with daytime urinary incontinence.	High
14	Deshmukh et al., 2024, India (3)	II trimester	no data (abortion)	none	none (abortion)	none (abortion)	none (abortion)	Moderate
15	Phillips et al., 2025, USA (19)	6 days of life	at 3 weeks of life	1. Amniocentesis	1. Peritoneal drainage 2. Urethral catheterization 3. PUV ablation 4. Circumcision	1. VCUG 2. US	no data	Moderate

### 3.3 Perinatal characteristics and timing of diagnosis

Gestational age was available in 13 cases. Term birth (≥37 weeks) was observed in 7/13 patients (54%).

Prenatal diagnosis was established in 5 of 15 patients (33%), most commonly during the third trimester (3/5; 60%), followed by the second trimester (2/5; 40%). In these cases, the diagnosis was based on US of large-volume fetal ascites with a collapsed or non-visualized bladder on subsequent examinations. No cases were diagnosed in the first trimester. In two cases, the diagnostic method was not reported: one pregnancy was terminated and one neonate died within a few hours after birth. Postnatal diagnosis occurred in 10 of 15 patients (66.7%), and in all of these cases bladder rupture was confirmed by VCUG. The diagnosis was made most frequently within the first two weeks of life (4/10; 40%), followed by weeks 2–4 of life (3/10; 30%). One case was diagnosed between 4 and 8 weeks of life, and delayed diagnosis was reported in one patient at 3 years of age. In one case, the exact timing of diagnosis was not specified. The median age at diagnosis for the entire cohort was 21 weeks of gestation.

### 3.4 Diagnostic evaluation

#### 3.4.1 Prenatal findings

Prenatal US findings were dominated by features of severe urinary tract obstruction. Fetal ascites was the most common finding, observed in 12/15 patients (80%), followed by hydronephrosis in 7/15 (47%). Bladder abnormalities were frequent, including bladder wall thickening in 7/15 patients (47%) and megacystis in 4/15 (27%). The bladder was undetectable in 2/15 patients (13%). A dilated posterior urethra (“keyhole sign”) was described in 2/15 patients (13%). Oligohydramnios was present in 7/15 cases (47%).

#### 3.4.2 Postnatal evaluation

Postnatal diagnostic data were available in 13/15 patients (87%), excluding two cases due to termination of pregnancy or early neonatal death. Bladder rupture was most commonly diagnosed postnatally by VCUG, which confirmed the diagnosis in 10 of 13 cases (76.9%). Renal and bladder ultrasound (US) was usually the initial imaging study and was performed in 9 of 13 patients (69%).

Additional imaging and functional studies included abdominal x-ray (1/13), 99mTc-dimercaptosuccinic acid renal scintigraphy (DMSA scan) (2/13), 99mTc-mercaptoacetyltriglycine scintigraphy (MAG3 scan) (1/13), chromium-51 ethylenediaminetetraacetic acid clearance (Cr-EDTA clearance) (1/13), magnetic resonance imaging (MRI) (1/13), intravenous pyelogram (IVP) (1/13), and video-urodynamics (1/13).

### 3.5 Interventions

#### 3.5.1 Prenatal interventions

Prenatal interventions were performed in 6/15 patients (40%). These included abdominal fetocentesis in 4/15 cases (27%), placement of a peritoneoamniotic shunt in 3/15 patients (20%), aspiration of urinomas in one patient, and amniocentesis in one case to exclude chromosomal abnormalities and fetal infections.

#### 3.5.2 Postnatal interventions

Postnatal management strategies were highly variable, reflecting heterogeneity in disease severity and clinical presentation. In 2/15 cases (13%), no postnatal intervention was undertaken due to termination of pregnancy or early neonatal death; therefore, subsequent percentage calculations are based on the intervention cohort (*n* = 13).

The most frequently performed definitive intervention was endoscopic ablation of PUV, carried out in 10/13 patients (77%) and in one patient a PUV resection was performed. Urethral catheterization after birth was used in 7/13 patients (54%), typically as an initial stabilization measure to ensure adequate urinary drainage while awaiting definitive treatment with valve ablation.

A significant proportion of patients required temporary urinary diversion or decompressive strategies. Vesicostomy was performed in 4/13 patients (31%), peritoneal drainage in 4/13 (31%), and suprapubic catheterization in 3/13 (23%). These findings highlight the frequent need for urgent decompression of the urinary tract in severe neonatal presentations.

Surgical repair of the bladder rupture were performed in 3/13 patients (23%). Antibiotic therapy was administered in 2/13 patients (15%), most commonly as adjunctive treatment in suspected or confirmed infection. A wide range of additional surgical interventions was observed (Table 1).

#### 3.5.3 Follow-up and outcomes

Follow-up data were available in 6/13 patients (46%), with reported durations ranging from 7 months to 8 years. No follow-up data were available for 7/13 patients (54%).

In the majority of patients with available follow-up, renal outcomes were favorable, characterized by stable kidney function and normal physical development, although isolated cases of persistent bladder dysfunction were observed (Table 1).

## 4 Discussion

Spontaneous prenatal bladder rupture is a rare and severe manifestation of PUV. It represents an extreme consequence of congenital lower urinary tract obstruction. Prenatal imaging findings usually reflect advanced obstruction and its sequelae. These may include megacystis, hydronephrosis, urinary ascites, a collapsed or non-visualized bladder on subsequent examinations, and oligohydramnios (4).

Bladder rupture should not be regarded as a benign event. Rather, it may be considered a marker of severe obstruction and a potential source of substantial bladder morbidity. Sudden decompression during fetal life may interfere with normal bladder development and contribute to a small-capacity, poorly compliant, or overactive bladder (1, 3). This was evident in our patient, who developed significant bladder dysfunction despite preserved kidney function during the available follow-up. Importantly, impaired bladder compliance, reduced capacity, or incomplete emptying may contribute to secondary upper urinary tract deterioration over time. Therefore, the clinical relevance of bladder rupture in PUV should be assessed not only in relation to early kidney outcomes, but also in terms of bladder function and the potential risk of later kidney deterioration.

Initial decompressive procedures, such as vesicostomy, reduce intravesical pressure and help protect the upper urinary tract, but they do not necessarily prevent subsequent bladder dysfunction (20). In several cases included in this review, management ultimately relied on permanent vesicostomy or another form of urinary diversion (9, 12, 18). Although kidney safety must remain the primary goal, patient comfort, independence, and daily functioning should also be considered. In our view, when feasible, bladder augmentation may provide a more physiological option by creating a larger, low-pressure reservoir. In our patient, ileocystoplasty combined with a continent catheterizable channel using the Macedo technique resulted in satisfactory functional outcomes. Nevertheless, this approach should be individualized and should not be regarded as a universal treatment pathway for all patients with PUV-associated bladder rupture.

In the setting of PUV, bladder rupture has recently been hypothesized to represent one of the possible “pop-off” phenomena. By allowing urinary extravasation into the peritoneal cavity, it may theoretically reduce intravesical and upper urinary tract pressure (4, 21). However, whether this pressure-relieving effect translates into preservation of kidney function remains unproven and cannot be inferred from the currently available evidence. Most available data come from single case reports with heterogeneous clinical presentations, variable management strategies, and very limited follow-up. Therefore, any association between bladder rupture and preserved kidney function should be interpreted cautiously.

In our patient, kidney function remained stable during the available follow-up despite severe prenatal obstruction. This observation is consistent with some previously reported cases, but it does not allow any conclusions regarding kidney protection. Importantly, early stabilization does not exclude later deterioration of kidney function. In our patient and in included studies the follow up period was too short too draw any conclusions. Several studies indicate that clinical problems in patients with PUV may become more evident during adolescence, when bladder demands increase and treatment adherence may become more challenging (22–25). Therefore, extended multidisciplinary care into adolescence and adulthood is essential to detect late complications and guide individualized management.

The present study has several limitations. The systematic review included only 15 reported patients. The available evidence is limited to single case reports. Clinical presentations, prenatal findings, diagnostic work-up, treatment strategies, and outcome measures were highly heterogeneous. Follow-up was short or incompletely reported. These limitations precluded quantitative synthesis and restrict the strength of any conclusions. As a result, the review should be interpreted as a descriptive summary of the available literature rather than evidence for a causal relationship between bladder rupture and kidney protection.

Overall, prenatal bladder rupture in PUV represents a rare and severe clinical entity. Although it has been hypothesized to act as an extreme pressure-relieving “pop-off” mechanism, this concept remains speculative. Available evidence is insufficient to determine whether bladder rupture provides meaningful kidney protection. At the same time, reported cases suggest that bladder rupture may be associated with significant bladder morbidity and, in selected patients, the need for complex reconstructive surgery.

## 5 Conclusion

Prenatal bladder rupture in posterior urethral valves is a rare and severe manifestation of congenital lower urinary tract obstruction. Although it has been proposed as a possible pressure-relieving “pop-off” mechanism, its protective role remains unproven and cannot be inferred from the currently available evidence.

The literature is limited to heterogeneous single case reports with incomplete follow-up. Therefore, conclusions regarding renal and bladder outcomes should remain cautious. Management should be individualized, with multidisciplinary follow-up focused on both kidney function and bladder development in this high-risk population.

## Data Availability

The original contributions presented in the study are included in the article/Supplementary Material, further inquiries can be directed to the corresponding author.
