# Correction: Fetal bladder rupture in posterior urethral valves: a clinically relevant complication or a protective pop-off mechanism

**DOI:** 10.3389/fped.2026.1937381

**Published:** 2026-07-27

**Authors:** Karolina Krzywiecka, Natalia Lekston, Zofia Sieroń, Hanna Kubik, Agnieszka Wiernik, Grzegorz Kudela

**Affiliations:** 1 Students’ Scientific Club at the Department of Pediatric Surgery and Urology, Faculty of Medical Sciences in Katowice, Medical University of Silesia, Katowice, Poland; 2 Department of Pediatric Surgery and Urology, Medical University of Silesia, Katowice, Poland

**Keywords:** bladder augmentation, pop-off mechanisms, posterior urethral valves, urinary ascites, urinary bladder rupture

Text correction

In the Methods section, within the description of the PICO framework, the labels “**Population:**” and “**Comparator:**” were inadvertently removed.

A correction has been made to the section:

2 Materials and methods, 2.2 Systematic review

“Pediatric patients, including fetal, neonatal, infant, and childhood cases, diagnosed with PUV complicated by bladder rupture, with or without urinary ascites.”

It should read: “Population: Pediatric patients, including fetal, neonatal, infant, and childhood cases, diagnosed with PUV complicated by bladder rupture, with or without urinary ascites.”

“Not applicable, as the available evidence consists predominantly of descriptive case reports and no control group was expected.”

It should read: "Comparator: Not applicable, as the available evidence consists predominantly of descriptive case reports and no control group was expected.”

The original article has been updated.

