# Supplementary material for: Fetal bladder rupture in posterior urethral valves: a clinically relevant complication or a protective pop-off mechanism
Source: Front Pediatr. 2026 Jul 10;14:1880245. doi: 10.3389/fped.2026.1880245 (PMC13395759; doi:10.3389/fped.2026.1880245)
Supplement: Supplementary file 1 [file Datasheet1.pdf]

Title: Fetal Bladder Rupture in Posterior Urethral Valves: A Clinically Relevant Complication or a Protective Pop-Off Mechanism

Authors: Krzywiecka Karolina, Lekston Natalia, Sieroń Zofia, Kubik Hanna, Wiernik Agnieszka, Kudela Grzegorz

Background

Posterior urethral valves (PUV) represent one of the most common causes of congenital bladder outlet obstruction in boys, leading to increased intravesical pressure and potential renal damage beginning in fetal life. In response, "pop-off" mechanisms may develop to decompress the urinary tract and potentially preserve renal function.

Spontaneous prenatal bladder rupture with urinary ascites is a rare and extreme manifestation of these mechanisms. While it may transiently reduce pressure, its long-term impact on bladder development and renal outcomes remains unclear. Existing evidence is limited to case reports and small case series, with no comprehensive synthesis of clinical presentation, management strategies, and outcomes.

Rationale

There is ongoing debate whether fetal bladder rupture in PUV represents:

- a protective mechanism (reducing pressure and preserving renal function), or

- a harmful complication (leading to bladder dysfunction and need for reconstructive surgery).

A systematic review is needed to synthesize available evidence and clarify its clinical implications.

Objective / Aim

To systematically review the literature on fetal or neonatal bladder rupture in patients with PUV, focusing on:

- clinical presentation and timing of diagnosis

- diagnostic findings

- prenatal and postnatal management strategies

- short- and long-term outcomes, particularly renal and bladder function

PICO Framework

1. Population (P)

Pediatric patients (prenatal, neonatal, or childhood) diagnosed with posterior urethral valves complicated by bladder rupture and/or urinary ascites

2. Intervention (I)

Prenatal interventions (e.g., fetocentesis, shunt placement)

Postnatal management (e.g., catheterization, valve ablation, urinary diversion, reconstructive surgery)

3. Comparator (C)

Not applicable (descriptive review; no control group expected)

4. Outcomes (O)

- Timing and mode of diagnosis
- Prenatal and postnatal imaging findings
- Types of interventions
- Complications
- Renal outcomes (including progression to renal failure)
- Bladder function and need for reconstructive surgery
- Survival

Eligibility Criteria

Inclusion Criteria

1. Studies reporting pediatric cases of bladder rupture (prenatal or postnatal) in the context of PUV
2. Case reports, case series, and observational studies
3. Studies with sufficient clinical and outcome data

Exclusion Criteria

1. Non-English publications
2. Conference abstracts, video materials, letters to the editor, and review articles
3. Studies with incomplete or duplicate data
4. Studies not clearly involving PUV-associated bladder rupture

Search Strategy

A comprehensive literature search will be conducted in:

- PubMed
- Embase
- Scopus
- Web of Science
- Cochrane Library

Search terms:

("posterior urethral valves" OR "PUV" OR "LUTO") AND ("bladder rupture" OR "urinary ascites" OR "urinoma" OR "pop-off")

No date restrictions will be applied.

Study Selection

Titles and abstracts will be screened independently by three reviewers.

Full-text articles will be assessed for eligibility.

Disagreements will be resolved by consensus or consultation with a fourth reviewer

Reference lists of included studies will be manually screened

Data Extraction

Data will be extracted into a predefined database and will include:

- Study characteristics (author, year, setting)
- Patient demographics (gestational age, age at diagnosis)
- Timing of diagnosis
- Prenatal and postnatal findings
- Diagnostic methods
- Interventions (prenatal and postnatal)
- Complications and outcomes (renal function, bladder function, survival)
- Risk of Bias Assessment

Risk of bias will be assessed independently by two reviewers using the Joanna Briggs Institute (JBI) critical appraisal checklist for case reports. Discrepancies will be resolved by consensus.

Given the predominance of descriptive study designs, the overall level of evidence is expected to be low and will be interpreted with caution.

Data Synthesis

Due to anticipated heterogeneity in study design and reporting:

- Data will be synthesized descriptively and narratively
- No meta-analysis is planned

Dissemination

Results will be published in a peer-reviewed journal and presented at scientific conferences.
